# Fermentation in Pineapple Juice Significantly Enhances Ornithine and Citrulline Production in *Lactococcus lactis* MSC-3G Isolated from Sugarcane

**DOI:** 10.3390/microorganisms10050962

**Published:** 2022-05-03

**Authors:** Yusuke Inoue, Narandalai Danshiitsoodol, Masafumi Noda, Katsushi Hagihara, Masanori Sugiyama

**Affiliations:** 1 Department of Probiotic Science for Preventive Medicine, Graduate School of Biomedical and Health Sciences, Hiroshima University, Hiroshima 8551, Japan; d201606@hiroshima-u.ac.jp (Y.I.); naraa@hiroshima-u.ac.jp (N.D.); bel@hiroshima-u.ac.jp (M.N.); 2 Mitsui Sugar Co., Ltd., Nihonbashi-Hakozakicho, Chuo-ku, Tokyo 103-8423, Japan; katsushi.hagihara@mitsui-sugar.co.jp

**Keywords:** *Lactococcus lactis*, ornithine, citrulline, arginine deiminase, fruit juice, sugarcane

## Abstract

Lactic acid bacterial (LAB) fermentation of functional amino acids using fruit juices as a cultivation medium is not well-documented. In the present study, we successfully isolated a high ornithine- and citrulline-producing *Lactococcus lactis* strain, designated MSC-3G, from sugarcane and investigated the ornithine and citrulline production profile using various fruit juices as a cultivation medium. Among fruit juices, pineapple juice exhibited the highest potentiality to initiate ornithine production (56 mM), while the highest citrulline yield was obtained during lime juice cultivation (34.5 mM). Under the optimal cultivation condition, the highest yield of ornithine and citrulline in pineapple juice reached 98.9 ± 2.2 mM and 211.1 ± 35.7 mM, respectively, both of which were significantly higher than that in the well-known industrial strain of *Corynebacterium* (*C*.) *glutamicum*. Additionally, citrulline production was dependent on oxygen supplementation and increased twofold when grown aerobically. Whole genome sequencing showed that the MSC-3G genome possesses the arginine deiminase (ADI) gene cluster arcABD1C1C2TD2. The results of the ADI pathway enzyme activities of MSC-3G showed a significant increase in arginine deiminase activity, while ornithine carbamoyl transferase activity was decreased, which in turn indicates the high citrulline-accumulation ability of MSC-3G when cultivated in pineapple juice.

## 1. Introduction

Lactic acid bacteria (LABs) are a diverse group of gram-positive, non-sporulating bacteria that have a long history of safe use and have therefore acquired Generally Recognized as Safe (GRAS) status when administrated in adequate amounts [1,2]. LABs have many benefits, notably as probiotics in human health and as bio-preservatives in the food industry. Due to their abundance in various environments, LABs exhibit significantly different functional properties with respect to the host organisms. All *Bifidobacterium* and some *Lactobacillus* strains permanently reside in different types of nutrient-rich mucosal cavities of a wide range of animals, constituting specific multifunctional microflora. Animal-derived LABs can assimilate lactose and are thus broadly used in dairy-product manufacturing. On the other hand, some LABs inhabit fastidious growth environments such as soil and the surface of plants. Particularly, as a minor part of endophytic bacteria, LABs that colonize symbiotically in plants can grow in low temperatures and are more aerotolerant than those originating in animals. As demonstrated in previous studies, plant-derived LABs often have genome-encoding enzymes that convert pentoses, cellobiose, sucrose, and malic and citric acids into other compounds [3]. Moreover, as described by Chopin [4], most *Lactococcus* (*L.*) lactis strains, which were isolated from plants, are prototrophic and do not require branched-chain amino acids (BCAAs) for their growth. Compared with the plant-derived *L. lactis* strains, the same species isolated from dairy products are auxotrophs due to the accumulated mutations and deletions within their BCAA biosynthetic gene cluster [5].

Functional amino acids have been thought to be associated with cell development, immune improvement, and the regulation of key metabolic pathways [6]. Ornithine produced in the urea cycle plays an important role as a hepatoprotective agent and converts excess ammonia to urea, which facilitates the metabolism of the tricarboxylic acid (TCA) cycle and gluconeogenesis. It has been demonstrated that ornithine promotes growth-hormone secretion, which eventually leads to the anti-fatigue effect and improves sleep and awakening, skin quality, and the development of muscle and bone [7,8]. Citrulline also presents in the ornithine cycle, and the assimilation of citrulline produces nitric oxide (NO) in the body, which dilates blood vessels and promotes blood flow [9]. Moreover, citrulline has been reported to have effects such as relief from arteriosclerosis and recovery from fatigue [10,11,12]. In animals, the biosynthetic pathway of these amino acids is regulated via the arginase pathway, whereas the metabolism of arginine in bacteria is regulated by either arginase or the arginine deiminase (ADI) pathway [13]. The ADI pathway plays a crucial role in protection of the bacteria against acidic or starved environmental conditions through the production of ATP and ammonia [14,15,16]. In general, the bacterial ADI pathway is composed of an arginine deiminase (arcA), which converts arginine to citrulline; an ornithine carbamoyl transferase (arcB), which converts citrulline to ornithine and carbamoyl phosphate; a carbamate kinase (arcC), which degrades carbamoyl phosphate to carbon dioxide and ammonia to produce ATP; and a potentiometric non-forming arginine/ornithine exchanger (arcD), which generates the intracellular uptake of arginine and the extracellular release of ornithine [17,18,19,20].

A limited number of studies have been reported with respect to ornithine and citrulline production by LABs. For example, our research group previously showed that the plant-derived *Weissella* (*W.*) *confusa* K-28 strain produced 18 ± 1 and 10 ± 2 g/L ornithine and citrulline, respectively, in the de Man, Rogosa, and Sharpe (MRS) medium supplemented with arginine. This strain harbors an ADI gene cluster, wkaABDCR [21]. Another report showed that the kimchi-derived *W*. *koreensis* DB1 produced 15.05 g/L ornithine and 6.2 g/L citrulline in an MRS medium supplemented with 3.0% arginine [22]. *Lactobacillus* (*L.*) *plantarum* Lp60, which is isolated from red wine, has also been reported to degrade arginine through the ADI system and produce 8.51 µmol citrulline/min/µg of protein and 1.89 µmol ornithine/min/µg of protein in a Niven medium [23]. Despite these strains converting arginine and producing a significant amount of ornithine and citrulline in a chemical medium, the practical significance of the functional amino acid production via the fruit juice–fermentation pathway remains unknown. Furthermore, due to the drawbacks of lactose intolerance and the high cholesterol content in dairy fermentation, we aimed to determine the ability of MSC-3G, a LAB strain isolated from sugarcane, to produce ornithine and citrulline in non-dairy fermentation, such as in a fruit juice medium, and to investigate the optimal condition for high-yield synthesis of these FAAs.

## 2. Materials and Methods

### 2.1. Isolation of an Ornithine-Producing LAB from Sugarcane

MRS medium (Becton, Dickinson and Company, Franklin Lakes, NJ, USA) was used to isolate and maintain the bacterial culture. Sugarcane samples were obtained from Kikaijima Island, Kagoshima Prefecture, and slices of stem, leaf, and roof parts were suspended in an MRS medium. After cultivation at 28 or 37 °C, culture of each isolate was spread on the MRS agar medium and incubated anaerobically at the given temperature for 2–3 days. Gram-staining, catalase-activity, and CO_2_ gas production tests were performed on the purified colony. A gram-staining test was performed using the Bermy M stain kit (Muto Pure Chemical Co., Ltd., Tokyo, Japan), and the cell morphology was observed under a microscope. Catalase activity was determined by a bubble test using 3% of hydrogen peroxide. The ornithine production by the isolated LAB strains was investigated by culturing for 72 h in the MRS medium supplemented with 1.0% (*w*/*v*) arginine.

### 2.2. DNA Sequencing and Identification of the LAB Strain

Identification of the isolated ornithine-producing LAB was performed using the entire 16S ribosomal DNA (rDNA) sequence analysis. For PCR, the genomic DNA was isolated from the LAB strain using a GenElute™ Bacterial Genomic DNA Kit (Sigma Aldrich, St. Louis, MO, USA). The 16S rDNA gene fragment was amplified by PCR using 27f (5′-AGAGTTTGATCCTGGCTAG-3′) and 1525r (5′-AAAGGAGGTGATCCAGCC -3′) primers. The PCR products were purified by using NucleoSpin Gel and PCR Clean-up kit (Macherey-Nagel GmbH and Co. KG, Duren, Germany), and the sequence of the fragment was determined using the method described previously [24,25]. Sequence similarity were performed using Basic Local Alignment Search Tool (BLAST) online at the National Centre for Biotechnology Information (NCBI) homepage (http://blast.ncbi.nlm.nih.gov/Blast.cgi, accessed on 17 April 2022). A whole genome sequence analysis of the MSC-3G strain was performed at the Oral Microbiome Center (Kagawa, Japan).

### 2.3. In Vitro Evaluation of Probiotic Characteristics

The fermentation potential of various sugars was confirmed using the API 50 CHL kit (bioMerieux, Lyon, France) according to the manufacturer’s instructions. A gastric acid-resistance test was conducted by using the 1st fluid for the disintegration test (Fujifilm Wako Pure Chemical Co., Ltd., Osaka, Japan), adjusted to pH 2.5 by NaOH as the artificial gastric juice. The LAB culture was anaerobically incubated with the artificial gastric juice at the final concentration of 1% *v*/*v*. After the incubation at 37 °C for 5 h, the living bacterial cells were counted and compared with those without the gastric juice. A total of 1% (*v*/*v*) of the LAB strain culture was inoculated into an MRS medium containing 0.1, 0.2, or 0.3% (*w*/*v*) bile acid powder, and the cells were statically incubated at 37 °C for 18 h. After the incubation, the OD_595_ of the cultures was measured and compared with that of the untreated ones.

### 2.4. Production of Ornithine and Citrulline in the Fruit Juice Medium

Pineapple, mango, orange, pear, muscat, grape, strawberry, lemon, lemon, lime, mandarin, yuzu, red grapefruit, white grapefruit, hyuganatsu fruit juice concentrates (Bussan Food Materials Co., Ltd., Tokyo, Japan) were used as media. Each concentrate was diluted with sterile deionized water to single-strength juice, and the pH of each fruit juice was adjusted to the specified pH with sodium hydrogen carbonate. A total of 1, 2, 3, 5, 7.5, 10, or 30% (*w*/*v*) arginine was added to the adjusted fruit juices and cultured at 20, 25, 28, 30, or 37 °C for 48–72 h. To determine the effect of the oxygen, each cultivation was performed under anaerobic, microaerobic, and aerobic conditions. The sampling was carried out 0, 24, 48, and 72 h after the start of culture, and the ornithine and citrulline amounts, bacterial counts, and pH of the culture medium were measured.

### 2.5. The HPLC Conditions for Ornithine, Arginine, Citrulline

The concentrations of arginine, ornithine, and citrulline were determined using the phenyl isothiocyanate (PITC) derivatization method [26]. A 10 µL culture supernatant sample was placed in a microcentrifuge tube and dried under reduced pressure. Then 20 µL of the mixed reagent (ethanol/water/trimethylamine (TEA) = 2/2/1) was added and mixed via vortex. After drying under reduced pressure, 20 µL of the mixed reagent (ethanol/water/TEA/PITC = 7/1/1/1) was added and kept at room temperature for 20 min. The reaction mixture was dried completely, dissolved in 1 mL of PTC-amino acid mobile phase A (Fujifilm Wako Pure Chemical Co., Ltd., Osaka, Japan), filtered with a 0.22 µm pore-size filter, and stored at −20 °C until use. For HPLC, a Wakopak^®^ Wakosil-PTC (4.0 mm × 250 mm, Fujifilm Wako Pure Chemical Co., Ltd., Osaka, Japan) was used. The mobile phase consists of a mixture of PTC-amino acid mobile phase A and PTC-amino acid mobile phase B (Fujifilm Wako Pure Chemical Co., Ltd., Osaka, Japan) in a linear gradient elution (0–20 min, 0–70% B). The column chromatography was performed at 40 °C with a flow rate of 1.0 mL/min under 254 nm.

### 2.6. Assay of Enzyme Activities in Cell Extracts

Cells cultured in fruit juice supplemented with arginine for 48 h were harvested and suspended into a 50 mM phosphate buffer (pH 7.5). The cell suspension was sonicated, and cell-free extracts were obtained. The protein concentrations of the cell-free extracts were measured using the TaKaRa Bradford Protein Assay Kit (Takara Bio Co., Ltd., Shiga, Japan). Arginine deiminase activity was assayed by measuring the rate of arginine change during the incubation for 40 min at 30 °C in a reaction mixture containing a 100 mM citrate buffer (pH 5.5) with 5 mM MnCl_2_, 5 mM L-arginine, and the cell extracts. Ornithine carbamoyl-transferase activity was assayed by measuring the conversion rate of ornithine changed during the incubation for 40 min at 30 °C of reaction mixtures containing a 150 mM imidazole buffer (pH 7.5) with 10 mM carbamoyl phosphate, 10 mM L-ornithine, 10 mM phosphate, and cell extracts. The concentration of arginine changed to ornithine was measured via HPLC.

## 3. Results and Discussions

### 3.1. Isolation and Carbohydrate Utilization of an Ornithine-Producing LAB Strain

We isolated and identified a total of twelve LAB candidates from sugarcanes in the present study. All strains were gram-positive, catalase-negative, and non-motile anaerobic bacteria. Among these strains, MSC-3G, which was identified as *L. lactis* in the 16S rDNA sequence analysis, exhibited high production of ornithine in the MRS medium supplemented with 5% arginine. The whole genome analysis demonstrates that the genome size of the MSC-3G strain is 2,481,272 bp and does not contain any plasmids. The genes encoding the ADI pathway in MSG-3G were clustered and encoded the arginine deiminase (arcA), ornithine carbamoyl transferase (arcB), carbamate kinase (arcC), arginine/ornithine antiporter (arcD), aminotransferase (arcT), arginine tRNA ligase (argS), and arginine repressor (argR) (Table 1).

This is consistent with the findings in previous studies, particularly the genetic structure of the ADI pathway cluster in the well-known international prototype *L. lactis* MG1363, which was isolated from milk. The gene cluster contains nine genes arranged in the following order: argR, argS, arcA, arcB, arcD1, arcC1, arcC2, arcT, and arcD2. Except for argR, all genes are transcribed in the same orientation, while argR, predicted to be involved in arginine-dependent regulation, is located upstream of argS and divergently transcribed [27,28].

Further, the profile of carbohydrate utilization, evaluated using the API 50 CHL test (Table S1), demonstrated that monosaccharides metabolized by MSG-3G include D-xylose, L-arabinose, D-galactose, D-fructose, D-glucose, D-mannose, and N-acetyl glucosamine. The utilization of disaccharide and polysaccharide by MSG-3G might indicate that it produces hydrolytic enzymes responsible for the cleavage of various sugars (α-glucosidase for trehalose, sucrose, and maltose; β-glucosidase for cellobiose and gentiobiose) and utilizes sugar alcohols (amygdalin, salicin, and mannitol). Carbon sources such as cellobiose, gentiobiose, and amygdalin are plant-derived carbohydrates and naturally occur in ripened fruit [29]. Further, MSC-3G has been shown to be resistant to acid stress (pH 2.5) and slightly tolerant of bile salt (0.1%).

### 3.2. Screening of Ornithine and Citrulline Production in Various Fruit Juices

**Figure 1:**
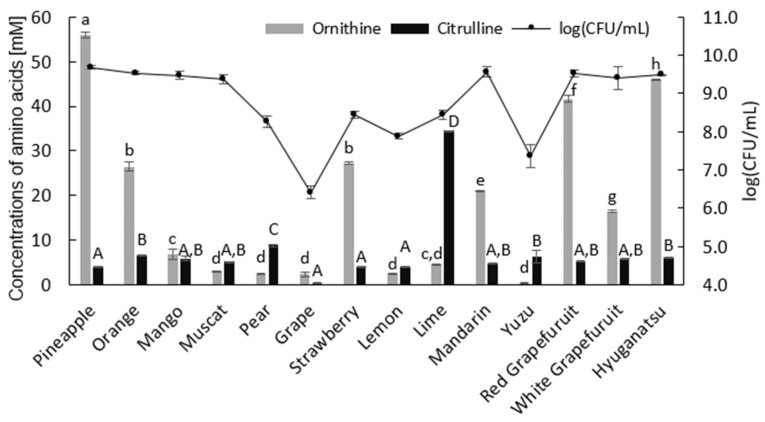
Ornithine and citrulline production of MSC-3G by the cultivation in each fruit juices supplemented with 1.0% (*w*/*v*) arginine. The cultivation was performed at pH 6.5 and 28 °C. All experiments were performed in triplicate. Means with different small letters show significant differences within ornithine production according to the Tukey HSD test at *p* ≤  0.01. Means with different capital letters show significant differences within citrulline production according to the Tukey HST test at *p* <  0.01. Error bars represent ± standard deviation.

A total of fourteen commercially available fruit juices were screened for the production of ornithine and citrulline (Figure 1).

Each juice fermentation of MSC-3G showed significantly different characteristics regarding the cell growth and production of ornithine and citrulline. Both cell growth and arginine metabolism were significantly lower when cultured in grape, lemon, and yuzu juices, while other juice cultivations showed cell levels between 8.3 and 9.7 log CFU/mL. Despite satisfactory cell growth, the arginine metabolism profiles were significantly different in each culture. The highest ornithine production (56.0 ± 0.7 mM) was obtained in pineapple juice, and the highest citrulline production was obtained in lime juice (34.5 ± 0.0 mM) even though the cell growth was somewhat lower in the lime culture than in the pineapple culture (8.4 vs. 9.7 logs CFU/mL, respectively). It is evident that each juice differed in its content of sugars, vitamins, organic acids, and other micronutrients, yet few literature data are available on the comparison of these nutrients. According to Li et al. [30], the sucrose and quinic acid contents were significantly higher in peach juice compared with apple, grape, and pear juices, while tartaric acid was detected only in grape juice. Another study of the sugar profiles in several fruit juices showed a high ratio of glucose and fructose for grape juices, whereas orange and mandarin juices had the ratio of less than one [31]. Fruit juices are also good source of various vitamins. It has been shown that orange, grapefruit, and pineapple juices contain a high amount of vitamin B complex; on the other hand, a large amount of vitamin C is present in orange, lemon, lime, mango juices [32,33]. Taken together, all these compounds and other micronutrients may show synergistic effects on arginine metabolism either directly or indirectly. According to the Food Composition Database, Japan, the manganese content is significantly higher in pineapple juice (1.16 g per 100 g of juice) than in other juices. Several studies have reported that the manganese ion is an important factor in the manganese catalase involved in oxidative stress tolerance [34,35,36]. Moreover, a research group showed that the addition of manganese ions to the fermentation enhanced lactate dehydrogenase (LDH) activity, which in turn resulted in a significant beneficial effect on fermentation of the LAB [37]. On the other hand, the increase in final cell growth in the mango juice cultivation (9.5 logs CFU/mL) inversely correlated to the ornithine and citrulline production (6.7 ± 0.7 and 5.8 ± 0.5 mM, respectively). Recently, Noens et al. [38] demonstrated that the substrate specificity of the l-arginine/l-ornithine exchangers ArcD1 and ArcD2 were significantly different in *L. lactis* MG1363. Even though both enzymes showed higher transport activities with l-arginine and l-ornithine, ArcD2 had a lower affinity to the cationic amino acids l-ornithine, l-lysine, and l-histidine. In contrast, ArcD2 showed a higher affinity to the neutral amino acid l-alanine and efficiently translocated l-alanine, while ArcD1 did not. Thus, the contradictory results in the mango juice fermentation might be due to the amino acid composition differences in the juices, since the nutritional compositions of mango juice showed that l-alanine content is 82 mg per 100 g of juice, which is significantly higher than that of pineapple juice.

### 3.3. Effects of the Cultivation Condition on Ornithine and Citrulline Productivity

We investigated the effects of the initial pH of the pineapple juice medium supplemented with 1% arginine with respect to the production of ornithine and citrulline (Table 2).

The growth rate of MSC-3G in the given initial pH medium was slightly different, but in general, it was positively correlated with the initial pH of rice. MSC-3G manifested exponential growth in the 24 h of fermentation at a pH range of 4.5 to 8.0, while the growth was slower at pH 4.0. The conversions of 1% arginine (*w*/*v*) to ornithine with an initial pH range of 4.5 to 8.0 were similar and reached their maximum conversion rate within 48 h of the cultivation. Meanwhile, an initial pH of 8.0 was found to be favorable for maximum ornithine and citrulline yield after 72 h of fermentation (32.2 ± 2.9 and 5.1 ± 1.6, respectively). The ADI pathway functions not only in the acquisition of energy sources but also in the response to cell growth under acid-stressed conditions [39]. Rimaux et al. [40,41] reported that arginine conversion through the ADI pathway in *L. sakei* CTC494 was influenced by the medium pH. In the medium pH range between 5.0 and 6.5, citrulline was converted to ornithine after the full depletion of arginine. On the other hand, the putative role of pH in regulation of the ADI pathway and the survival of *L. sakei* RV1000 during the stationary growth phase was investigated. An LDH-deficient mutant that does not produce lactate, and thus does not lower the pH of the medium below 6.20, showed a significant growth decrease during the stationary phase when arginine was omitted from the broth. However, when arginine was added to the medium, the final pH of the 48 h cultivation reached 8.35 due to the degradation of arginine and cell viability increased [42].

In the present study, we also investigated the suitable culture temperature to obtain higher ornithine and citrulline production (Table 3).

Interestingly, arginine was almost completely converted to ornithine after 24 h when cultured under a temperature lower than 30 °C, while citrulline production reached 2.8 ± 0.7 mM at 37 °C. The ornithine productivity agreed with our previous study, where the optimal temperature to convert arginine to ornithine in *W*. *confusa* K-28 was lower than 30 °C [21]. It was demonstrated that the sourdough-derived *L. fermentum* IMDO130101 possesses an environmental pH, and temperature affected the ADI pathway. The ratio between ornithine and citrulline and the formation pattern varied as a function of temperature. The conversion of citrulline from ornithine increased with an increase in temperature [43].

**Figure 2:**
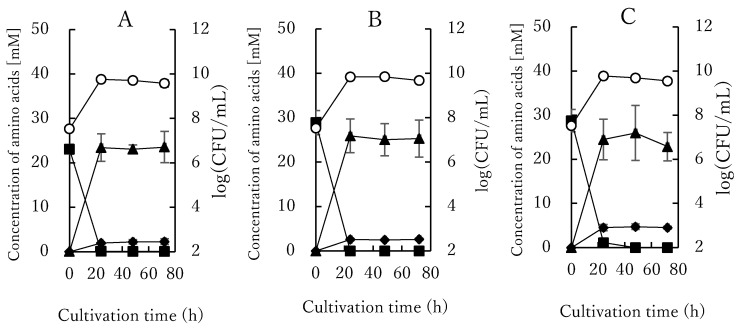
Difference of ornithine and citrulline production and cell growth under the aerobic or microaerobic or anaerobic condition. The cultivation was performed at pH 6.5 and 28 °C using the medium supplemented with 1.0 (*w*/*v*) % arginine. Growth (log (CFU/mL)); ○ of *L. lactis* MSC-3G and extracellular concentrations (µM) of arginine (■), citrulline (◆), and ornithine (▲) at different O_2_ conditions ((**A**) anaerobic; (**B**) microaerobic; (**C**) aerobic). Full lines represent the predictions by the model; symbols represent the experimental data. The data were expressed as mean ± S.E. (*n* = 3).

To further investigate the role of oxygen in arginine metabolism, we compared anaerobic, microaerophilic, and aerophilic fermentation of MSC-3G in pineapple juice supplemented with 1.0 (*w*/*v*) % arginine (Figure 2).

The growth rate was similar in each condition, but only a marginal increase was observed under microaerophilic and aerophilic conditions compared with anaerobic conditions. On the other hand, at periodic intervals of cultivation, the estimated citrulline production of MSC-3G increased with the fermentation time, and the concentration of citrulline increased from 2.2 ± 0.5 mM with anaerobic fermentation to 4.7 ± 0.6 mM with aerobic fermentation. In our previous study, gene expression of the ADI pathway of *W. confusa* K-28 was shown to be higher under anaerobic conditions than under an aerobic one [21], and this is consistent with the theory that multiple prokaryotic lineages use the ADI pathway as an energy source under an anaerobic condition. Conversely, there are very few reports in which the increase in arginine metabolism under an aerobic condition has been discussed, especially on *Pseudomonads*. The argR from *Pseudomonas aeruginosa* represses the carbamoyl phosphate synthetase and anabolic ornithine carbamoyl transferase but mediates induction of the aruCFGDBE operon that encodes the arginine aminotransferase pathway (ATA), a major arginine metabolic pathway under aerobic conditions [44]. Yet in other LAB strains no increased citrulline production under aerobic conditions has been described.

### 3.4. The Relationship between the Ratio of Conversion to Ornithine and the Concentration of Arginine Added to the Medium

To evaluate the effect of arginine supplementation on ornithine production by MSC-3G experiments were performed in which 1, 2, 3, 5, 7.5, 10, and 30% (*w*/*v*) L-arginine was added to the pineapple juice before cultivation (Table 4).

We observed that either the growth rate or the ornithine and citrulline production was affected by the different arginine concentrations. With the addition of arginine up to 7.5%, the final growth rate was similar at periodic intervals of cultivation (from 9.3 ± 0.1 to 9.86 ± 0.04 CFU/mL), and the highest ornithine production was observed during 48 h cultivation supplemented with 7.5% arginine (98.9 ± 2.2 mM). Both the arginine depletion and the cell growth of MSC-3G were higher in cultures with lower arginine concentrations and gradually decreased with increasing initial concentrations of arginine supplementation. Particularly, the remaining arginine in the medium after the stationary phase of growth was significantly higher when the medium was supplemented with more than 10% arginine. Concerning the effect of supplemented arginine on cell growth and conversion, studies revealed that *L. sanfranciscensis* CB1 fermented in sourdough completely converted arginine with 6 mM, while higher concentrations of arginine did not show any effect on the lactic acid or ethanol production of the strain [45]. As for citrulline, its production by MSC-3G during fermentation reached a maximum value of 211.1 ± 35.7 mM at 72 h of cultivation supplemented with 10% arginine; however, the cell growth was markedly decreased to 8.4 ± 0.1 CFU/mL. It has been indicated that the addition of a high concentration of arginine to *L. buchneri* CUC-3 and *Oenococcus* (*O.*) *oeni*, the main LABs in winemaking, enhanced the growth rate of *L. buchneri* but not *O. oeni* [46,47]. The same was also observed in *L. plantarum* Lp60, where the cell growth was reduced when the arginine concentration increased from 0.4 g/L to 2 g/L [23]. Additionally, Hwang et al. [48] reported that, compared with the other LAB species isolated from the kimchi, the *L. lactis* strain did not show increased citrulline production, and the ornithine content was lower when cultivated in an MRS medium supplemented with arginine.

### 3.5. Assay of ADI Pathway Enzyme Activities in Cell Extracts

The bacterial ADI pathway involves three catalytic enzymes: an arginine deiminase (EC 3.5.3.6), an ornithine carbamoyl transferase (EC 2.1.3.3), and a carbamate kinase (EC 2.7.2.2). In fact, arginine depletion, efficiency, and the ratio of the end products greatly depend on the activity of these enzymes and transports. Therefore, we measured the enzymatic activities of MSC-3G cultivated in pineapple and mango juices with and without arginine supplementation. The activities of the arginine deiminase and carbamoyl transferase were evaluated using the conversion rates of arginine and ornithine, respectively (Table 5).

The fermentation of pineapple juice without the arginine supplement resulted in a 39.5% arginine conversion. However, the conversion rate increased approximately twofold (79%) when arginine was added. In the case of mango juice, the highest conversion rate without arginine was 16.8%, while the conversion rate with arginine supplementation was only 25.8%. Moreover, the concentration of total protein of the MSC-3G strain in the cell changed from 0.43 mg/mL without arginine to 0.64 mg/mL in pineapple juice supplemented with arginine, which is almost three times higher than that in mango juice. Under the conditions used, the carbamoyl transferase activities of MSC-3G also were measured. In the fermentation of pineapple juice without arginine the conversion rate of arginine to ornithine was 46.3%, and this rate increased to 59% with the addition of arginine. On the other hand, the ornithine conversion rate in mango juice supplemented with arginine was 30.2%. It should be noted that the mango juice fermentation revealed a higher arginine deiminase assay correlated with a higher carbamoyl transferase assay. Meanwhile, the fermentation of pineapple juice by the MSC-3G strain enhanced the arginine deiminase activity and reduced the carbamoyl transferase activity when arginine was added to the medium. Another group demonstrated that *L. plantarum* Lp60 accumulates citrulline in the medium, as shown by its high arginine deiminase activity and low ornithine carbamoyl-transferase activity [23]. Thus, it can be concluded that the arginine deiminase activity of MSC-3G is significantly higher in pineapple juice than that in mango juice, and the formation of end-product amino acids depends on the activity ratio of the correlating enzymes.

## 4. Conclusions

In this study, we isolated high ornithine- and citrulline-producing *L. lactis* strain MSC-3G from sugarcane and determined the rate of ornithine and citrulline production using various fruit juices as media. To increase the productivity of both amino acids, the effects of factors such as the initial pH, cultivation temperature, oxygen supply, and arginine supplementation were studied. We found that the ADI pathway enzymes on the pineapple juice medium supplemented with arginine exhibited higher activity than those on mango juice, and these activities are likely caused by the juice nutrient components.

## Figures and Tables

**Table 1:** The ADI gene cluster in *L. lactis* MSC-3G.

Gene	aa	Best Blast Homology (Source)	Accession No. of Related Protein	Identity (%)
arcD2	497	Arginine/Ornithine antiporter (*L. lactis subsp. lactis* IL1403)	Q9CE19	99.2
arcT	376	Aminotransferase (*L. lactis subsp. lactis* IL1403)	Q9CE18	98.9
arcC2	314	Carbamate kinase (*L. lactis subsp. lactis* IL1403)	Q9CE17	99.4
arcC1	314	Carbamate kinase (*L. lactis subsp. lactis* IL1403)	Q9CE16	99.0
arcD1	490	Arginine/Ornithine antiporter (*L. lactis subsp. lactis* IL1403)	Q9CE15	99.4
arcB	354	Ornithine carbamoyl transferase (*L. lactis subsp. lactis* IL1403)	P0C2U2	99.7
arcA	410	Arginine deiminase (*L. lactis subsp. lactis* IL1403)	P58013	100.0
argS	1695	Arginine tRNA ligase (*L. lactis subsp. lactis* IL1403)	Q9CE12	99.8
argR	459	Arginine repressor (*L. lactis subsp. lactis* IL1403)	Q9CE11	96.1

**Table 2:** Effects of the initial pH of the pineapple juice medium supplemented with 1% of arginine.

Initial pH	Cultivation Time (Hour)	Amino Acid Concentrations (mM)			log (CFU/mL)
		Arg	Orn	Cit	
4.0	0	30.0 ± 2.6	0.1 ± 0.1	0.0 ± 0.0	7.53 ± 0.04
	24	28.4 ± 0.3	7.4 ± 2.1	0.3 ± 0.1	7.9 ± 0.3
	48	10.1 ± 5.0	21.9 ± 5.2	0.7 ± 0.7	8.2 ± 0.5
	72	7.4 ± 3.6	26.2 1.1	0.7 ± 0.7	7.9 ± 0.5
4.5	0	24.8 ± 1.6	0.1 ± 0.1	0.0 ± 0.0	7.53 ± 0.04
	24	0.1 ± 0.1	26.0 ± 1.7	2.0 ± 0.6	9.60 ± 0.04
	48	0.1 ± 0.1	27.2 ± 0.9	1.9 ± 0.7	9.6 ± 0.1
	72	0.1 ± 0.1	22.0 ± 0.6	1.9 ± 0.8	9.4 ± 0.1
5.0	0	26.7 ± 0.4	0.1 ± 0.1	0.0 ± 0.0	7.53 ± 0.04
	24	0.1 ± 0.1	23.3 ± 3.7	1.9 ± 0.3	9.80 ± 0.05
	48	0.1 ± 0.1	24.1 ± 2.7	2.0 ± 0.5	9.6 ± 0.1
	72	0.1 ± 0.1	24.4 ± 2.4	1.8 ± 0.4	9.3 ± 0.1
5.5	0	26.0 ± 0.6	0.1 ± 0.1	0.0 ± 0.0	7.53 ± 0.04
	24	0.1 ± 0.1	23.9 ± 2.2	1.9 ± 0.3	9.8 ± 0.1
	48	0.1 ± 0.1	23.9 ± 0.7	2.1 ± 0.7	9.76 ± 0.04
	72	0.1 ± 0.1	21.8 ± 0.8	1.8 ± 0.5	9.5 ± 0.1
6.0	0	26.9 ± 0.6	0.1 ± 0.1	0.0 ± 0.0	7.53 ± 0.04
	24	0.1 ± 0.1	25.1 ± 3.3	1.9 ± 0.1	9.8 ± 0.1
	48	0.1 ± 0.1	26.0 ± 2.2	3.2 ± 1.4	9.7 ± 0.1
	72	0.1 ± 0.1	23.0 ± 1.7	2.0 ± 0.5	9.68 ± 0.04
6.5	0	23.1 ± 1.1	0.1 ± 0.1	0.0 ± 0.0	7.53 ± 0.04
	24	0.1 ± 0.1	23.4 ± 3.1	1.9 ± 0.2	9.76 ± 0.03
	48	0.1 ± 0.1	23.1 ± 1.0	2.2 ± 0.5	9.71 ± 0.04
	72	0.1 ± 0.1	23.6 ± 3.5	2.3 ± 0.7	9.6 ± 0.2
7.5	0	30.4 ± 6.3	0.0 ± 0.0	0.0 ± 0.0	7.53 ± 0.04
	24	0.0 ± 0.0	28.0 ± 3.1	3.3 ± 0.2	9.7 ± 0.1
	48	0.0 ± 0.0	26.4 ± 2.2	3.3 ± 0.3	9.79 ± 0.01
	72	0.0 ± 0.0	31.0 ± 3.5	3.4 ± 0.1	9.57 ± 0.01
8.0	0	30.9 ± 3.5	0.0 ± 0.0	0.0 ± 0.0	7.53 ± 0.04
	24	0.0 ± 0.0	30.7 ± 3.9	4.8 ± 2.0	9.78 ± 0.04
	48	0.0 ± 0.0	30.8 ± 2.9	4.9 ± 1.5	9.86 ± 0.02
	72	0.0 ± 0.0	32.2 ± 2.9	5.1 ± 1.6	9.74 ± 0.04
8.5	0	23.3 ± 3.9	0.0 ± 0.0	0.0 ± 0.0	7.53 ± 0.04
	24	1.4 ± 0.2	19.9 ± 4.5	2.0 ± 0.3	9.8 ± 0.2
	48	0.0 ± 0.0	26.4 ± 3.6	1.5 ± 0.1	9.87 ± 0.02
	72	0.0 ± 0.0	27.2 ± 2.5	1.6 ± 0.1	9.74 ± 0.01

The data were expressed as mean ± S.E. (*n* = 3).

**Table 3:** Effects of the cultivation temperature of the pineapple juice medium supplemented with 1% of arginine.

Temp. (°C)	Cultivation Time (Hour)	Amino Acid Concentrations (mM)			log (CFU/mL)
		Arg	Orn	Cit	
20	0	24.8 ± 2.8	0.1 ± 0.1	0.0 ± 0.0	7.51 ± 0.03
	24	0.1 ± 0.1	22.4 ± 0.3	1.3 ± 0.4	9.7 ± 0.1
	48	0.1 ± 0.1	24.6 ± 0.7	1.4 ± 0.5	9.9 ± 0.1
	72	0.1 ± 0.1	23.9 ± 0.7	1.3 ± 0.4	9.8 ± 0.1
25	0	24.8 ± 2.8	0.1 ± 0.1	0.0 ± 0.0	7.51 ± 0.03
	24	0.1 ± 0.1	25.7 ± 0.8	1.9± 0.8	9.70 ± 0.04
	48	0.1 ± 0.1	24.6 ± 2.0	0.7 ± 0.4	9.80 ± 0.04
	72	0.1 ± 0.1	24.5 ± 2.5	0.6 ± 0.4	9.80 ± 0.03
28	0	23.1 ± 1.1	0.1 ± 0.1	0.0 ± 0.0	7.51 ± 0.03
	24	0.1 ± 0.1	23.4 ± 3.1	1.9 ± 0.2	9.76 ± 0.03
	48	0.1 ± 0.1	23.1 ± 1.0	2.2 ± 0.5	9.71 ± 0.04
	72	0.1 ± 0.1	23.6 ± 3.5	2.3 ± 0.7	9.6 ± 0.2
30	0	24.7 ± 2.7	0.1 ± 0.1	0.0 ± 0.0	7.51 ± 0.03
	24	0.1 ± 0.1	24.3 ± 0.4	2.1 ± 0.6	9.75 ± 0.03
	48	0.1 ± 0.1	25.6 ± 1.1	2.4 ± 0.1	9.73 ± 0.03
	72	0.1 ± 0.1	22.1 ± 0.9	2.2 ± 0.9	9.65 ± 0.02
37	0	23.2 ± 1.3	0.1 ± 0.1	0.0 ± 0.0	7.51 ± 0.03
	24	0.1 ± 0.1	20.8 ± 0.2	2.7 ± 0.7	9.6 ± 0.1
	48	0.1 ± 0.1	21.9 ± 0.1	2.8 ± 0.7	9.6 ± 0.3
	72	0.1 ± 0.1	20.2 ± 1.2	2.5 ± 0.8	5.5 ± 0.2

The data were expressed as mean ± S.E. (*n* = 3).

**Table 4:** Effects of supplemented arginine concentrations in the pineapple juice medium on the ornithine and citrulline production and cell growth.

Arg (% (*w*/*v*))	Cultivation Time (Hour)	Amino Acid Concentrations (mM)			Log (CFU/mL)
		Arg	Orn	Cit	
1.0	0	28.5 ± 4.3	0.0 ± 0.0	0.0 ± 0.0	7.5 ± 0.1
	24	0.1 ± 0.1	23.3 ± 2.6 ^a,b^	6.2 ± 2.6 ^A,C^	9.7 ± 0.1
	48	0.1 ± 0.1	25.6 ± 3.8 ^a^	5.5 ± 0.1 ^A^	9.7 ± 0.1
	72	0.1 ± 0.1	24.7 ± 3.3 ^a,e^	5.7 ± 0.1 ^A^	9.7 ± 0.1
2.0	0	50.7 ± 0.7	0.0 ± 0.0	0.0 ± 0.0	7.49 ± 0.03
	24	0.1 ± 0.1	41.7 ± 2.8 ^b^	6.2 ± 0.9 ^A,C^	9.75 ± 0.03
	48	0.1 ± 0.1	38.5 ± 3.5 ^a^	5.5 ± 0.8 ^A^	9.80 ± 0.01
	72	0.1 ± 0.1	40.4 ± 4.5 ^a,b^	5.7 ± 0.9 ^A^	9.86 ± 0.04
3.0	0	73.4 ± 1.5	0.0 ± 0.0	0.0 ± 0.0	7.49 ± 0.03
	24	1.8 ± 0.8	50.5 ± 5.7 ^b,c^	12.0 ± 1.5 ^A,C^	9.8 ± 0.1
	48	0.1 ± 0.1	56.9 ± 0.9 ^b^	14.9 ± 1.4 ^A^	9.83 ± 0.054
	72	0.1 ± 0.1	53.8 ± 5.0 ^b,c^	14.6 ± 1.8 ^A^	9.83 ± 0.04
5.0	0	129.4 ± 21.0	0.0 ± 0.0	0.0 ± 0.0	7.5 ± 0.1
	24	67.5 ± 11.6	73.6 ± 8.5 ^c^	11.3 ± 2.6 ^A,C^	9.7 ± 0.1
	48	5.3 ± 3.8	81.2 ± 2.8 ^c^	36.3 ± 2.8 ^A^	9.71 ± 0.04
	72	0.0 ± 0.0	75.4 ± 1.0 ^d^	39.3 ± 4.5 ^A^	9.7 ± 0.1
7.5	0	214.9 ± 6.0	0.0 ± 0.0	0.0 ± 0.0	7.5 ± 0.0
	24	144.6 ± 11.6	61.1 ± 5.5 ^b,c^	71.2 ± 1.6 ^B^	9.40 ± 0.01
	48	44.8 ± 13.9	98.9 ± 2.2 ^c^	161.1 ± 8.0 ^B^	9.3 ± 0.1
	72	0.0 ± 0.0	86.9 ± 6.5 ^d^	184.8 ± 8.6 ^B^	9.5 ± 0.1
10.0	0	268.6 ± 58.4	0.0 ± 0.0	0.0 ± 0.0	7.5 ± 0.1
	24	257.9 ± 42.8	7.2 ± 5.3 ^a^	26.1 ± 13.1 ^A^	8.7 ± 0.1
	48	174.6 ± 8.2	33.7 ± 7.9 ^a^	123.6 ± 26.0 ^B^	8.69 ± 0.03
	72	73.5 ± 19.5	42.6 ± 9.9 ^a,b^	211.1 ± 35.7 ^B^	8.4 ± 0.1
30.0	0	956.1 ± 82.3	0.0 ± 0.0	0.0 ± 0.0	7.5 ± 0.1
	24	870.1 ± 54.4	0.0 ± 0.0 ^a^	0.0 ± 0.0 ^C^	3.6 ± 0.1
	48	865.1 ± 55.3	0.0 ± 0.0 ^d^	0.0 ± 0.0 ^A^	3.57 ± 0.04
	72	887.6 ± 68.5	0.0 ± 0.0 ^e^	0.0 ± 0.0 ^A^	3.55 ± 0.03

The data were expressed as mean ± S.E. (*n* = 3). Means with different small letters show significant differences within ornithine production according to the Tukey HSD test at *p* ≤  0.05. Means with different capital letters show significant differences within citrulline production according to the Tukey HST test at *p* <  0.05.

**Table 5:** Total protein concentrations and enzyme activities of the ADI pathway.

Juice	Arg	Protein Concentrations (mg/mL)	Conversion Rates of Arginine (%)	Conversion Rates of Ornithine (%)
Pineapple	-	0.43 ± 0.04	39.5 ± 5.8	46.3 ± 1.5
	+	0.64 ± 0.05	79.0 ± 2.7	59.0 ± 2.4
Mango	-	0.16 ± 0.02	16.8 ± 3.2	18.1 ± 0.3
	+	0.23 ± 0.07	25.8 ± 2.4	30.2 ± 1.5

The data were expressed as mean ± S.E. (*n* = 3).

## Data Availability

The data presented in the study are available in article.

## Supplementary Materials

The following supporting information can be downloaded at: https://www.mdpi.com/article/10.3390/microorganisms10050962/s1, Table S1: Carbohydrate utilization profile of MSC-3G.

## References

[1] Sanders M.E. (2008). Probiotics: Definition, sources, selection, and uses. Clin. Infect. Dis..

[2] Rattanachaikunsopon P., Phumkhachorn P. (2020). Lactic acid bacteria: Their antimicrobial compounds and their uses in food production. Ann. Biol. Res..

[3] Nomura M., Kobayashi M., Narita T., Kimoto-Nira H., Okamoto T. (2006). Phenotypic and molecular characterization of *Lactococcus lactis* from milk and plants. J. Appl. Microbiol..

[4] Chopin A. (1993). Organization and regulation of genes for amino acid biosynthesis in lactic acid bacteria. FEMS Microbiol. Rev..

[5] Godon J.J., Delorme C., Bardowski J., Chopin M.C., Ehrlich S.D., Renault P. (1993). Gene inactivation in *Lactococcus lactis*: Branched-chain amino acid biosynthesis. J. Bacteriol..

[6] Tamura T., Noda M., Ozaki M., Maruyama M., Matoba Y., Kumagai T., Sugiyama M. (2010). Establishment of an efficient fermentation system of Gamma-aminobutyric acid by a Lactic Acid Bacterium, *Enterococcus avium* G-15, isolated from carrot leaves. Biol. Pharm. Bull..

[7] Ho Y.Y., Nakato J., Mizushige T., Kanamoto R., Tanida M., Akiduki S., Ohinata K. (2017). l-Ornithine stimulates growth hormone release in a manner dependent on the ghrelin system. Food Funct..

[8] Miyake M., Kirisako T., Kokubo T., Miura Y., Morishita K., Okamura H., Tsuda A. (2014). Randomized controlled trial of the effects of L-ornithine on stress markers and sleep quality in healthy workers. Nutr. J..

[9] Curis E., Nicolis I., Moinard C., Osowska S., Zerrouk N., Bénazeth S., Cynober L. (2005). Almost all about citrulline in mammals. Amino Acids.

[10] Schwedhelm E., Maas R., Freese R., Jung D., Lukacs Z., Jambrecina A., Spickler W., Schulze F., Böger R.H. (2008). Pharmacokinetic and pharmacodynamic properties of oral L-citrulline and L-arginine: Impact on nitric oxide metabolism. Br. J. Clin. Pharmacol..

[11] Hayashi T., Juliet P.A., Matsui-Hirai H., Miyazaki A., Fukatsu A., Funami J., Iguchi A., Ignarro L.J. (2005). l-Citrulline and l-arginine supplementation retards the progression of high-cholesterol-diet-induced atherosclerosis in rabbits. Proc. Natl. Acad. Sci. USA.

[12] Takeda K., Machida M., Kohara A., Omi N., Takemasa T. (2011). Effects of citrulline supplementation on fatigue and exercise performance in mice. J. Nutr. Sci. Vitaminol..

[13] Cunin R., Glansdorff N., Pierard A., Stalon V. (1986). Biosynthesis and metabolism of arginine in bacteria. Microbiol. Rev..

[14] Fang F., Zhang J., Zhou J., Zhou Z., Li T., Lu L., Zeng W., Du G., Chen J. (2018). Accumulation of citrulline by microbial arginine metabolism during alcoholic fermentation of soy sauce. J. Agric. Food Chem..

[15] Araque I., Bordons A., Reguant C. (2013). Effect of ethanol and low pH on citrulline and ornithine excretion and arc gene expression by strains of *Lactobacillus brevis* and *Pediococcus pentosaceus*. Food Microbiol..

[16] Araque I., Reguant C., Rozes N., Bordons A. (2011). Influence of wine-like conditions on arginine utilization by lactic acid bacteria. Int. Microbiol..

[17] Barcelona-Andrés B., Marina A., Rubio V. (2002). Gene structure, organization, expression, and potential regulatory mechanisms of arginine catabolism in *Enterococcus faecalis*. J. Bacteriol..

[18] Majsnerowska M., Noens E.E.E., Lolkema J.S. (2018). Arginine and citrulline catabolic pathways encoded by the arc gene cluster of *Lactobacillus brevis* ATCC 367. J. Bacteriol..

[19] Fulde M., Willenborg J., Huber C., Hitzmann A., Willms D., Seitz M., Eisenreich W., Weigand P.W., Goethe R. (2014). The arginine-ornithine antiporter ArcD contributes to biological fitness of *Streptococcus suis*. Front. Cell. Infect. Microbiol..

[20] Vrancken G., Rimaux T., Weckx S., De Vuyst L., Leroy F. (2009). Environmental pH determines citrulline and ornithine release through the arginine deiminase pathway in *Lactobacillus fermentum* IMDO 130101. Int. J. Food Microbiol..

[21] Rakhimuzzaman M., Noda M., Danshiitsoodol N., Sugiyama M. (2019). Development of a system of high ornithine and citrulline production by a plant-derived lactic acid bacterium, *Weissella confusa* K-28. Biol. Pharm. Bull..

[22] Yeong M.S., Hee M.S., Choon C.H. (2020). Characterization of high-ornithine-producing *Weissella koreensis* DB1 isolated from kimchi and its application in rice bran fermentation as a starter culture. Foods.

[23] Spano G., Massa S., Arena M.E., de Nadra M.C.M. (2007). Arginine metabolism in wine *Lactobacillus plantarum*: In vitro activities of the enzymes arginine deiminase (ADI) and ornithine transcarbamylase (OTCase). Ann. Microbiol..

[24] Hiraishi A. (1992). Direct automated sequencing of 16S rDNA amplified by a polymerase chain reaction from bacterial cultures without DNA purification. Lett. Appl. Microbiol..

[25] Weisburg W.G., Barns S.M., Pelletier D.A., Lane D.J. (1991). 16S ribosomal DNA amplification for phylogenetic study. J. Bacteriol..

[26] Heinrikson R.L., Meredith S.C. (1984). Amino acid analysis by reverse-phase high-performance liquid chromatography: Precolumn derivatization with phenylisothiocyanate. Anal. Biochem..

[27] Larsen R., Buist G., Kuipers O.P., Kok J. (2004). ArgR and AhrC are both required for regulation of arginine metabolism in *Lactococcus lactis*. J. Bacteriol..

[28] Budin-Verneuil A., Maguin E., Auffray Y., Ehrlich S.D., Pichereau V. (2006). Genetic structure and transcriptional analysis of the arginine deiminase (ADI) cluster in *Lactococcus lactis* MG1363. Can. J. Microbiol..

[29] Buckenhüskes H.J., Doyle P.D., Beuchat L.R., Montville T.J. (1997). Fermented vegetables. Food Microbiology: Fundamentals and Frontiers.

[30] Li J., Zhang C., Liu H., Liu J., Jiao Z. (2020). Profiles of sugar and organic acid of fruit juices: A comparative study and implication for authentication. J. Food Qual..

[31] Navarro-Pascual-Ahuir M., Lerma-García M.J., Simo-Alfonso E.F., Herrero-Martínez J.M. (2015). Rapid differentiation of commercial juices and blends by using sugar profiles obtained by capillary zone electrophoresis with indirect UV detection. J. Agric. Food Chem..

[32] Dillon A., Nagy S., Wade R.L. (1995). Fruit juice profiles. Methods to Detect Adulteration of Fruit Juice Beverages.

[33] Wills R.B.H. (1987). Composition of Australian fresh fruits and vegetables. Food Technol. Aust..

[34] Archibald F. (1986). Manganese: Its acquisition by and function in the lactic acid bacteria. Crit. Rev. Microbiol..

[35] Archibald F.S., Fridovich I. (1981). Manganese and defenses against oxygen toxicity in *Lactobacillus plantarum*. J. Bacteriol..

[36] Watanabe M., van der Veen S., Nakajima H., Abee T. (2012). Effect of respiration and manganese on oxidative stress resistance of *Lactobacillus plantarum* WCFS1. Microbiology.

[37] Cheng X., Dong Y., Su P., Xiao X. (2014). Improvement of the fermentative activity of lactic acid bacteria starter culture by the addition of Mn^2+^. Appl. Biochem. Biotechnol..

[38] Noens E.E., Kaczmarek M.B., Żygo M., Lolkema J.S. (2015). ArcD1 and ArcD2 arginine/ornithine exchangers encoded in the arginine deiminase pathway gene cluster of *Lactococcus lactis*. J. Bacteriol..

[39] Casiano-Colón A., Marquis R.E. (1988). Role of the arginine deiminase system in protecting oral bacteria and an enzymatic basis for acid tolerance. Appl. Environ. Microbiol..

[40] Rimaux T., Vrancken G., Pothakos V., Maes D., Vuyst L.D., Leroy F. (2011). The kinetics of the arginine deiminase pathway in the meat starter culture *Lactobacillus sakei* CTC 494 are pH-dependent. Food Microbiol..

[41] Rimaux T., Rivière A., Illeghems K., Weckx S., De Vuyst L., Leroy F. (2012). Expression of the arginine deiminase pathway genes in *Lactobacillus sakei* is strain-dependent and is affected by the environmental pH. Appl. Environ. Microbiol..

[42] Verges M.C., Zúñiga M., Morel-Deville F., Pérez Martínez G., Zagorec M., Ehrlich S.D. (1999). Relationships between arginine degradation, pH and survival in *Lactobacillus sakei*. FEMS Microbiol. Lett..

[43] Vrancken G., Rimaux T., Wouters D., Leroy F., Vuyst L.D. (2009). The arginine deiminase pathway of *Lactobacillus fermentum* IMDO 130101 responds to growth under stress conditions of both temperature and salt. Food Microbiol..

[44] Lu C.D., Winteler H., Abdelal A., Haas D. (1999). The ArgR regulatory protein, a helper to the anaerobic regulator ANR during transcriptional activation of the arcD promoter in Pseudomonas aeruginosa. J. Bacteriol..

[45] De Angelis M., Mariotti L., Rossi J., Servili M., Fox P.F., Rollán G., Gobbetti M. (2002). Arginine catabolism by sourdough lactic acid bacteria: Purification and characterization of the arginine deiminase pathway enzymes from *Lactobacillus sanfranciscensis* CB1. Appl. Environ. Microbiol..

[46] Mira De Orduña R., Patchett M.L., Liu S.Q., Pilone G.J. (2001). Growth and arginine metabolism of the wine lactic acid bacteria *Lactobacillus buchneri* and *Oenococcus oeni* at different pH values and arginine concentrations. Appl. Environ. Microbiol..

[47] Tonon T., Lonvaud-Funel A. (2000). Metabolism of arginine and its positive effect on growth and revival of *Oenococcus oeni*. J. Appl. Microbiol..

[48] Hwang H., Lee J.H. (2018). Characterization of arginine catabolism by lactic acid bacteria isolated from kimchi. Molecules.

