# Supplementary material for: Fermentation in Pineapple Juice Significantly Enhances Ornithine and Citrulline Production in Lactococcus lactis MSC-3G Isolated from Sugarcane
Source: Microorganisms. 2022 May 3;10(5):962. doi: 10.3390/microorganisms10050962 (PMC9143541; doi:10.3390/microorganisms10050962)
Supplement: Supplementary file 1 [file microorganisms-10-00962-s001.zip › microorganisms-1709479-supplementary.pdf]

Table S1. Carbohydrate utilization profile of MSC-3G.

Characteristic	Results
Carbon source fermentability	
Glycerol	-
Erythritol	-
D-Arabinose	-
L-Arabinose	+
D-Ribose	+
D-Xylose	+
L-Xylose	-
D-Adonitol	-
Methyl-βD-xylopyranoside	-
D-Galactose	+
D-Glucose	+
D-Fructose	+
D-Mannose	+
L-Sorbose	-
L-Rhamnose	-
Dulcitol	-
Inositol	-
D-Mannitol	+
D-Sorbitol	-
Methyl-αD-mannopyranoside	-
Methyl-αD-glucoyranoside	-
N-Acetyl glucosamine	+
Amygdalin	+
Arbutin	+
Esculin ferric citrate	-
Salicin	+
D-Cellobiose	+
D-Maltose	+
D-Lactose	+
D-Melibiose	-
D-Sucrose	+
D-trehalose	+
Inulin	-

D-Melezitose	-
D-Raffinose	-
Starch	+
Glycogen	-
Xylitol	-
Gentiobiose	+
D-turanose	-
D-Lyxose	-
D-Tagarose	-
D-Fucose	-
D-Arabitol	-
Gluconate	+
2-Ketogluconate	-
5-Ketogluconate	-
